# Neuromyelitis optica spectrum disorder with massive basal ganglia involvement: a case report

**DOI:** 10.1186/s12883-019-1580-3

**Published:** 2019-12-30

**Authors:** Shinji Ohara, Taka-aki Miyahira, Kenya Oguchi, Yo-ichi Takei, Fumihiro Yanagimura, Izumi Kawachi, Kiyomitsu Oyanagi, Akiyoshi Kakita

**Affiliations:** 1 Department of Neurology, Matsumoto Medical Center, Minami 2-20-30, Matsumoto, 399-8701 Japan; 2 Department of Neurology, Iida Hospital, 1-15 Ohdori, Iida, 395-8505 Japan; 3 Department of Neurology, Brain Research Institute, Niigata University, Niigata, Japan; 4 Division of Neuropathology, Brain Research Center, Shinshu University School of Medicine, Matsumoto, Japan; 5 Brain Research Laboratory, Hatsuishi Hospital, Chiba, Japan; 6 Department of Neuropathology, Brain Research Institute, Niigata University, Niigata, Japan

**Keywords:** Neuromyelitis optica spectrum disorder (NMOSD), Astrocytopathy, Basal ganglia, Blood brain barrier

## Abstract

**Background:**

Occurrence of basal ganglia involvement in neuromyelitis optica spectrum disorders (NMOSD) has rarely been reported and none documented pathologically.

**Case presentation:**

A 73-year-old female was clinically diagnosed with a NMOSD based on the clinical and radiological features and positive serum autoantibodies to AQP4. One month before her death, she became acutely ill with disturbed consciousness and right hemiparesis, and was diagnosed and treated as having basal ganglia infarction based on the brain CT. She made a partial recovery but later died from heart failure. At autopsy, the corresponding basal ganglia process revealed a large fresh area of necrosis. Histologically, several pathological signatures of NMOSD could be recognized in the lesion, including inflammatory cell infiltrations by B and T lymphocytes, perivascular complement and fibrinogen deposition, and the appearance of numerous phagocytosed corpora amylacea within the infiltrating macrophages.

**Conclusions:**

The present case illustrates that basal ganglia may be directly involved in the pathological processes of NMOSD, although the possibility of modification of the lesions by superimposed regional ischemia could not be excluded.

## Background

Neuromyelitis optica spectrum disorder (NMOSD) is a severe inflammatory autoimmune disease of the central nervous system (CNS) associated with episodes of transverse myelitis, optic neuritis and other neurologic manifestations. Antoantibodies to the water channel aquaporin-4 (AQP4), which is predominantly expressed in astrocyte foot processes, is a serum biomarker and is expressed in a majority of the cases with this syndrome [1].

Neuropathologically, NMOSD has been regarded as an autoimmune astrocytopathy, in which vasculocentric inflammatory cell inflammation and complement deposition are cardinal features [2]. This astrocytopathy results in the formation of necrotic lesions typically in the spinal cord and in the optic tracts, often associated with macroscopic cavity formation [3]. Other CNS areas than spinal cord and optic tracts could also be involved in NMOSD, such as the cerebral hemisphere, internal capsule, and periventricular AQP4 enriched regions including the area postrema and hypothalamus [1, 3, 4]. However, direct involvement of the deep cerebral central gray matter especially of basal ganglia has rarely been documented.

We here report a patient with NMOSD who developed a large basal ganglia lesion 1 month prior to her death, which was initially diagnosed as an ischemic infarction. However, autopsy revealed unequivocal immunohistological features of NMO in the basal ganglia lesion.

### Case presentation

**Fig. 1:**
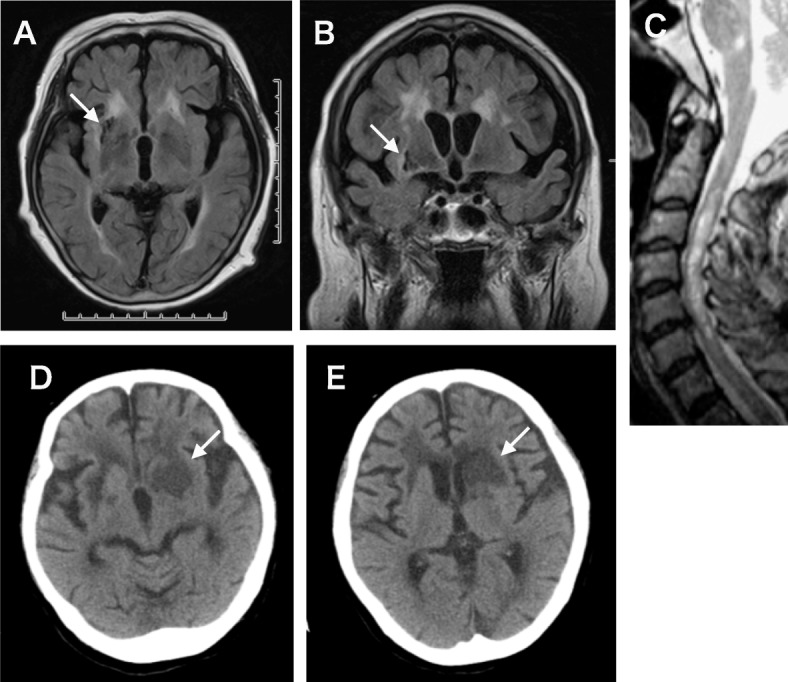
Radiologic findings. **a**-**c**) Brain MRI images taken 3 years after the onset revealed a cystic lesion in the right basal ganglia (arrow), which was subsequently histologically examined at autopsy (Fig. 2a, j). **a**) T2 MRI, **b**) Flair MRI. **c**) T2-MRI of the spinal cord taken 4 years after the onset, revealing longitudinal lesions involving C3–6 spinal segments. **d**) Brain CT taken 1 month prior to the death, a few days after the patient developed right hemiparesis and disturbed consciousness. The left basal ganglia show extensive low densities accompanied by narrowing of the lateral vetricles (arrow)

**Fig. 2:**
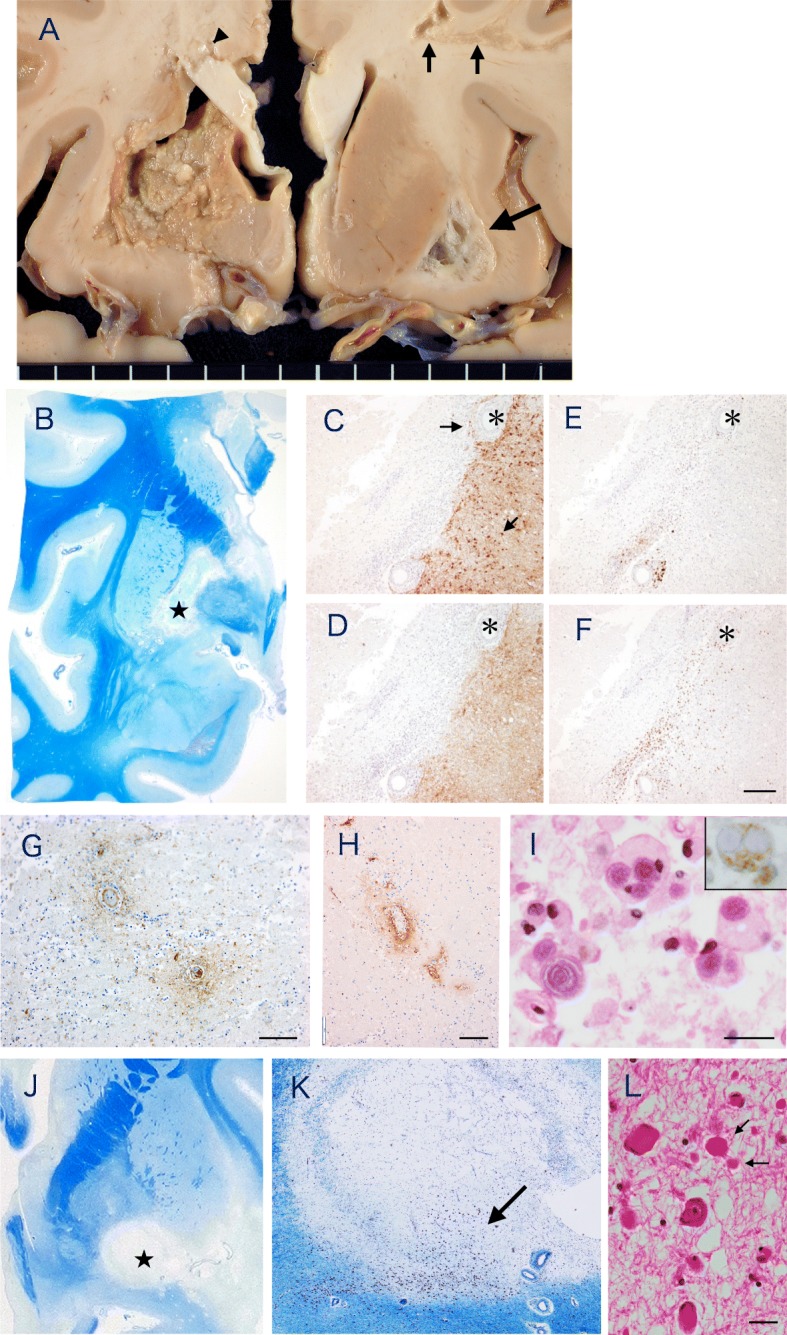
Neuropathology of the basal ganglia. **a** Coronal section of the brain after fixation with formalin. There is a large soft necrotic lesion involving the basal ganglia and a part of the internal capsule. A separate small necrotic focus is seen in the corpus callosum (arrowhead). In the right cerebral hemisphere, a large cystic lesion in the white matter lateral to the putamen (large arrow) and lesions in the subcortical white matter (small arrows) are seen. The optic chiasm appeared atrophic and there was a mild to moderate atherosclerotic changes in the basal arteries. **b** Klüver-Barrera myelin stain of the section approximately 1 cm posterior to. The necrotic area is well demarcated involving the globus pallidus extending to the internal capsule to the head of the caudate. **c**-**f** Serial sections stained immunohistochemically using the antibody for GFAP (**c**), AQP4 (**d**), CD138 for plasma cells (**e**) and CD8 for the marker of cytotoxic T cells (**f**). The necrotic lesions lacked immunostaining of both GFAP and AQP4 with a tendency to more preservation of the former (arrows in C). There are extensive CD138 and CD8 positive lymphocytic cell infiltrations in vasocentric patterns around small and medium sized vessels surrounding the area of necrosis. Bar = 200 μm. **g**-**i** Immunohistochemistry of the necrotic lesion stained for C9neo (**g**), fibrinogen (**h**), H.E. (**i**) and CD68 for macrophage (Inset of I). There were scattered foci of vasocentric complement deposition (**g**) and exudation of fibrinogen around the vessel wall (**h**). There are numerous macrophages containing corpora amylacea in the cytoplasm(I), as identified as such by the lack of immunostaining for CD68 (Inset of I). Bar = 100 μm (**g**, **h**), 20 μm (I, Inset). **j**-**l** Right basal ganglia lesions stained with Klüver-Barrera (**j**). There are well demarcated cystic lesions in the basolateral of the basal ganglia (*). **k** Enlarged area of (*) in (**j**). Microscopically, the lesion consisted of loosely associated fibrillary astrocytosis with few inflammatory cells. Klüver-Barrera myelin stain. **l** Enlarged area indicated by the arrow in (**k**). The neurons appeared relatively well preserved in the loosely arranged myelinated fibers. Arrows indicate axonal spheroid bodies. H&E. Bar = 20 μm

A previously healthy 63 year-old Japanese female presented with gait disturbance and was diagnosed as having transverse myelopathy with a sensory level of T5 and bilateral Babinski signs. She was given intravenous methyl prednisolone, resulting in a complete clinical recovery. At age 67, she started to complain of numbness on the left side of her face and developed an ataxic gait. MRI of the brain and the spinal cord revealed a focal lesion in the pontine tegmentum on the right, a cystic lesion in the subcortical white matter lateral to the basal ganglia on the right (Fig. 1a, b), and a longitudinally extending cavitary lesion affecting the C2-C6 cervical cord (Fig. 1c). Cerebrospinal fluid (CSF) examination revealed normal protein content (43 mg/dl) with increased myelin basic protein (MBP) (253 pg/ml, normal < 120). Oligoclonal bands were negative. Steroid therapy resulted in a good recovery. At age 69, she developed optic neuritis bilaterally. Serum antibodies to AQP4 was positive, and she was diagnosed as having NMOSD. After intravenous steroid therapy, she was started on a low maintenance dose of oral prednisone of 15 mg. At age 70, she was unable to walk and was admitted to our hospital. The neurologic examination revealed the patient had normal mental status, bilaterally decreased vision, mild dysarthria, weakness and sensory loss in the right upper extremity, and spastic paraplegia. Steroid administration was not effective in reversing her condition and she became wheelchair bound. At age 72, she developed acute myocardial infarction which was successfully treated with a stent placement. She was incidentally found to have aplastic anemia and received blood transfusions. One month prior to death, she developed right hemiparesis and disturbed consciousness over several days, and the brain CT revealed a large low-density lesion involving the left basal ganglia (Fig. 2d, e). A diagnosis of acute infarction was made and she was treated conservatively. She gradually recovered consciousness but never returned to her baseline. She died from heart failure at age 73.

The general autopsy was granted and was performed 1 hour after the death, which revealed old and recent myocardial infarcts and aspergillosis of the lung.

There was no evidence of systemic vasculitis. The brain was small and weighed 1080 g. There were mild atheromatous changes in the basal arteries of Willis. The bilateral optic nerves and chiasma were atrophic.

On the coronal sections, there was a large soft necrotic focus involving the putamen, globus pallidus, internal capsule and caudate nucleus on the left side (Fig. 2a). A soft necrotic lesion was also seen in the corpus callosum (arrowhead in Fig. 2a). On the opposite side of the cerebral hemisphere, there were cystic lesions in the subcortical and deep white matter (Fig. 2a. Thin and thick arrows). In the brainstem, there was a focal plaque-like lesions in the medial lemniscus. The spinal cord revealed central cavitary lesions extending from C3 to C5.

Formalin-fixed, paraffin embedded sections of the brain and the spinal cord were stained with Hematoxylin and Eosin (H&E), Klüver-Barrera (KB) and Grocott methenamine silver staining for fungi. Immunohistochemistry with a streptavidin-Biotin method was performed with the following antibodies. Rabbit polyclonal antibodies against AQP4 (Chemicon, 1:1000), glial fibrillary acidic protein (GFAP; DAKO, 1:800), myelin basic protein (MBP 1:150, Novocastra), the cytotoxic T-cell marker CD8 (1:50, DAKO), the plasma cell marker CD138 (1:200, DAKO), macrophage/microglial marker CD68 (1:100, DAKO), fibrinogen (1:3000, DAKO), C9neo (Dr. Paul Morgan, UK, 1:200). Reaction products were visualized with DAB.

Histologically, optic nerves revealed moderate gliosis without active features of neuronal degeneration. The wall of the small vessels in the optic nerve invariably showed hyalinized thickening. The necrotic foci in the left ganglia stained pale with H&E and KB stains, and was well demarcated and irregular in contour (Fig. 2b). The lesion was largely GFAP and AQP4 negative, with some tendency of relatively more GFAP preservation than AQP4 (Fig. 2c, d). At the margin surrounding the lesion, there was a prominent perivascular inflammatory cell infiltration around small and medium sized vessels. Many of the infiltrating cells were immunoreactive either for B and T lymphocyte or plasma cell markers (Fig. 2e, f). Immunohistochemistry with antibodies for C9neo and fibrinogen were positive in vasocentric patterns, which was found exclusively in the necrotic lesion (Fig. 2g, h). Numerous macrophages were also found to contain corpora amylacea in their cytoplasm (Fig. 2i). The Grocott methenamine silver stain was negative in the necrotic lesions. Occasional eosinophils and neutrophils could be recognized in the macroscopically fresh appearing lesion of the corpus callosum.

By contrast, the cystic lesions contralateral hemispheres were lined by fibrillary gliosis and there were few inflammatory cells (Fig. 2j-l). In the periphery of a lesion, many viable looking neurons were seen within the loosely arranged myelinated fibers (Fig. 2k, l). No thrombotic occlusions could be found in the lenticulostriate arteries on both sides despite an intensive search. There were no cortical demyelinating lesions except for several scattered foci of ischemic infarcts of various stage.

In the brain stem, the pontine tegmentum showed a few foci of gliosis associated with demyelination and relatively preserved axons. In the spinal cord, there were cystic lesions predominantly involving the central gray matter of the thoracic cord. There was relative sparing of axons compared to myelin in the area surrounding the cystic lesions, where the loss of AQP4 was much broader than those area with GFAP loss. In and around the cystic lesions, small vessels with thickened hyalinized walls were seen. There were only a few infiltrating T and B cells.

## Discussion and conclusion

The present case could be unequivocally diagnosed as NMOSD based on typical clinical course and imaging features, and a presence of positive serum AQP4 antibody [1, 5], except that, one-month pre-mortem, the patient developed consciousness disturbance and hemiparesis, which was considered to be due to a large basal ganglia infarct. However, upon autopsy, the affected basal ganglia revealed immunohistological signatures of NMO, such as perivascular deposition of active complement component and extensive vasocentric inflammatory cell infiltration consisting of T and B lymphocytes and plasma cells [2], a tendency of relative preservation of GFAP compared with AQP4 in the margin of the necrotic lesions [3]. Furthermore, numerous phagocytosed corpora amylacea were observed in the necrotic lesion. Corpora amylacea are age-dependent spherical, basophilic, PAS positive inclusions that are predominantly found in the astrocytic foot processes with certain predilections including around small vessels [6], and their appearance in phagocytosed state has been regarded as a histological hallmark of astrocytic injury in NMO [7]. It is speculated that corpora amylacea liberated from the degenerating astrocytic foot processes around blood vessels could be engulfed by infiltrating macrophages through intact regional blood supply [7].

Occurrence of basal ganglia involvement in NMOSD has been rarely reported in the literature [8, 9] and none documented pathologically. Therefore, one may wonder if NMO pathology could solely be responsible for causing such large area of necrosis in the basal ganglia as seen in the present patient. In this regard, it should be noted that the presence of AQP4 antibody alone does not exhibit any pathological effects in animals and humans [10, 11]. Nishiyama et al. suggested that certain immunologic events that increase in the Blood brain barrier (BBB) permeability are needed to allow entry and binding to AQP4 on astrocytes [11]. Recently, however, Jueneman et al. reported that in a rat stroke model, the animal infused with AQP4 antibodies suffered more vasogenic edema formation as compared to the controls, and that the size of the infarct was larger in the AQP4 antibody infused animals [12].

In the present case, exudation of fibrinogen could be demonstrated immunohistochemically around small vessels in the necrotic lesion, indicating the occurrence of BBB breakdown prior to the development of the necrotic lesion. The BBB anatomically consists of both the endothelial cells lining the capillary lumen and the surrounding astrocytic foot processes [13]. Moreover, it seems worthy of note that Ikuta et al. studied ultrastructural changes of astrocytes in various acute model of brain injuries including ischemia, and found that the first stage is characterized by swelling of the astrocytic foot processes surrounding the vessels and neurons, followed by the detachment of the astrocytic processes from vessel wall, suggesting the occurrence of BBB breakdown in the very acute stage of ischemia [14]. Therefore, it is possible to consider that pre-existing vasculopathy and/or focal ischemia may have been contributory to the acceleration of AQP4 antibody-mediated astrocytopathy through leaky BBB, resulting in the formation of a large necrotic lesions in the basal ganglia.

Because the basal ganglia are a common site of ischemic stroke especially with elderly patients with preexisting vasculopathy, its involvement in NMOSD may have been under-diagnosed so far. Further clinicopathological studies are required to elucidate the frequency and the nature of basal ganglia lesions in NMOSD.

## Data Availability

The datasets used in the current study are available form the corresponding author on reasonable request.

## Abbreviations

AQP4: Aquaporin 4
BBB: Blood brain barrier
CSF: Cerebrospinal fluid
GFAP: Glial fibrillary acidic protein
H.E.: Hematoxylin-eosin stain
MRI: Magnetic resonance imaging
NMO: Neuromyelitis optica
NMOSD: Neuromyelitis optica spectrum disorders
PAS: Periodic acid—Schiff stain
